# Dietary patterns in relation with nutritional outcomes and associated factors among adolescents: implications for context-specific dietary intervention for the Agrarian Community, Northwest Ethiopia

**DOI:** 10.3389/fnut.2023.1274406

**Published:** 2023-11-02

**Authors:** Eskezyiaw Agedew, Zeweter Abebe, Abebe Ayelign

**Affiliations:** Center for Food Science and Nutrition, College of Natural and Computational Sciences, Addis Ababa University, Addis Ababa, Ethiopia

**Keywords:** dietary patterns, nutrition outcomes, adolescents, Ethiopia, context-specific

## Abstract

**Introduction:**

Dietary pattern analysis allows us to characterize the dietary intakes of individuals rather than nutrient intake data and strongly predicts disease risks. The relationship between food intake and adolescents’ nutritional health is not well understood yet. Therefore, this study aimed to generate evidence for context-specific dietary intervention for adolescents.

**Objective:**

This study aimed to determine dietary patterns and their relationship with nutritional outcomes and identify the contributing factors among adolescents in the Agrarian Community of Northwest Ethiopia.

**Methods:**

A cross-sectional survey was conducted among 622 randomly selected adolescents. Dietary data were collected over a 1-week recall period using the Food Frequency Questionnaire (FFQ). After testing the basic assumptions, an exploratory factor analysis was conducted to determine the dietary patterns. Anthropometric data on weight and height were collected to determine the nutritional status using WHO Anthroplus 2010 software. A chi-square test was conducted to evaluate the effect of different dietary patterns on nutritional outcomes. A multivariable binary logistic regression model was used to identify factors affecting the dietary patterns of adolescents.

**Result:**

Three types of dietary patterns, namely, traditional, mixed, and animal-source foods with traditional alcoholic beverage consumption were identified. These dietary patterns explain 58.64% of the variance in adolescent diet in the study setting. The burden of stunting was 15.12% vs. 11.21, 19.40% vs. 6.94, and 8.36% vs.17.97% among adolescents with lower traditional, mixed, and higher animal sources with alcoholic dietary pattern consumption habits, respectively (value of *p* <0.05). Adolescents who resided in low-land agroecology (AOR = 2.44; 95% CL: 1.24, 4.81) and had access to animal-source foods (AOR = 1.64; 95% CI: 1.04, 2.60) were associated with lower consumption of traditional dietary patterns. Similarly, adolescents who resided in low-land (AOR = 1.80; 95% CI: 1.18, 2.74) had formal education (AOR = 2.38; 95% CI: 1.35, 4.19) and had poor nutrition knowledge (AOR = 2.83; 95% CL: 1.55, 5.19) were associated with lower consumption of mixed dietary patterns. Moreover, adolescents residing in the high-land (AOR = 2.50; 95% CI: 1.37, 4.56) and being female (AOR =1.87; 95% CI: 1.27, 2.74) were significant factors associated with lower consumption of animal-sourced foods with traditional alcoholic beverage consumption patterns.

**Conclusion:**

Multidimensional modifiable factors were explored that could be targeted for public health interventions for the identified dietary patterns. Integrated and multifaceted dietary intervention approaches are needed to promote healthy diets and discourage the consumption of unhealthy diets to reduce undernutrition in the study area and similar settings.

## Introduction

Adolescence is a transitional period characterized by the rapid growth and development that are the foundation for later life, health, and wellbeing. At this time, adolescents require essential nutrients such as protein, calcium, and iron for optimal growth and development (1, 2). This rapid growth rate combined with a lower nutrient intake leads to a risk of nutritional vulnerability and deficiencies in this age group (3).

The dietary pattern of an individual is sustainable nutrition behaviors to enhance the overall health of adolescents (1, 2). It helps to measure the number, proportion, variety, or combination of different foods and beverages in a more comprehensive way. This approach considers the interactions between foods and nutrients to promote health or reduce disease risk (4, 5).

Dietary pattern analysis of the target population helps to predict the risk of disease better than the analysis of isolated nutrients because the joint effect of the various nutrients involved would be better identified (5). It helps to understand the relationship between dietary habits and disease outcomes, which is important for public health actions to develop and provide diet-based intervention programs (6).

Unhealthy dietary patterns developed during adolescence can lead to diet-related chronic non-communicable diseases in later life (7, 8). Adolescents consume diets that are not in line with the recommended healthy diet; only 17% of adolescents have a diversified diet at the global level (9). This poor nutritional habit of adolescents leads to stunting, which exposes them to concurrent and future adverse health outcomes (3).

Adolescents from developed countries such as the United States, Europe, and Australia had low consumption habits of healthy diets such as fruits, vegetables, dairy products, and whole grains but higher consumption of unhealthy diets such as soft drinks and fast foods (10, 11). This problem also exists in adolescents living in low- and middle-income countries, where their eating habits are characterized by unhealthy habits (1, 2, 6, 12).

Adolescents had poor dietary habits in Ethiopia (13). According to a study conducted in Northwest Ethiopia, 32.30% of the adolescents had adequate dietary diversity, 97.70% of adolescent girls consumed starchy staples, 42.6% had no fruit intake, and only 1.70% of them consumed animal-source food (9, 14).

The burden of stunting and thinness among adolescents in Ethiopia ranges 12.50–33.10% and 12.60–58.30%, respectively (15–18). This figure indicates that undernutrition among adolescents is a significant health problem (19, 20). Adolescents’ dietary habits were influenced by various socioeconomic factors, such as lack of exposure to nutrition education and influence from family, peers, and the media. Access to and availability of diversified food items were the most commonly reported factors (2, 21).

Adolescents provide a second opportunity for nutritional interventions to mitigate all forms of nutritional deficiency (22). Hence, there is a need for updated evidence on dietary consumption patterns for designing and implementing appropriate nutrition interventions targeting adolescents. Previous studies have mainly focused on determining nutritional status and dietary diversity score assessments of adolescents, which did not consider the complexity of diets (23–26).

There is a lack of evidence to investigate dietary patterns and their relationship with nutrition outcomes among adolescents. Therefore, this study was conducted to generate evidence for public health interventions for adolescents’ nutritional interventions.

## Materials and methods

### Study approach and setting

A cross-sectional survey was conducted among adolescents who resided in six randomly selected areas of Dembecha Woreda, Northwest Ethiopia. Data were collected from 10 December 2021 to 20 January 2021. The study area consists of three agroecology zones (low-, mid-, and high-lands), which are conducive to diversified agricultural production.

Adolescents aged 10–19 years were the target population for the current study. These populations are overlooked for nutritional intervention in developing countries, including Ethiopia, as well as in the current setting. Adolescents with spinal curvature, who could not stand properly or walked with a wheelchair, did not participate in the study.

### Sample determination and approach

A single population proportion formula was used to determine the total number of adolescents in this study, based on the following assumptions: a 5% error margin, a 95% confidence interval, and a proportion of 44.6% for the consumption habit (P) of dark green leafy vegetables in southwest Ethiopia (25) with a design effect of 1.5. After adding a 10% non-response rate, the final sample size was 627.

The study area was purposefully selected due to its unique three agroecology zones (low-, mid-, and high-lands), which represent the northwest region of the country. Then, from each agroecology zone, two research settings, or Kebeles (the lowest administrative unit in the case of Ethiopia), were randomly selected as study areas for data collection.

Subsequently, from a randomly selected study setting, eligible households with at least one adolescent were registered, and a sampling frame was prepared. The number of adolescents assigned is proportional to the size (population) of a particular site. Finally, 357 adolescents from high-land, 77 from mid-land, and 188 from low-land agroecology zones were interviewed.

### Data collection tools and methods

A pretested, structured interviewer-administered questionnaire was used for data collection. The tool was adapted from peer-reviewed articles and customized to the local context. Data on the dietary patterns of adolescents were collected using a validated food frequency questionnaire (FFQ) and contextualized to the setting over 1 week. To develop the FFQ assessment tool, a list of local food items that were consumed at different times during meals and snacks was collected by conducting a pilot study on 30 adolescents for 2 non-consecutive weekdays and 1 weekend day (27). The FFQ was organized in a semi-quantitative manner and administered using 24-h dietary recall methods.

Anthropometric data on adolescents’ weight and height were measured using a standard weight and height scale with precisions of ±0.1 kg and ± 0.1 cm, respectively, based on WHO protocol (28). During measurement, adolescents wore lightweight clothing without shoes to minimize error. Trained human nutrition graduates collected data through interviews and anthropometric measurements.

### Data quality control

Data quality was ensured in all phases of the research activities. The tool was translated from English into an Amharic version (the local language). Training was provided to data collectors on the sampling procedures and interview techniques. The questionnaire was pretested using 5% of the total sample size at one site, which was not included in the actual study. In addition, before conducting actual dietary data collection from each sample, the FFQ was tested for internal reliability in 30 adolescents. The test results showed Cronbach’s alpha coefficient (α) of 0.79.

This result indicates that the FFQ is reliable for measuring the dietary patterns of adolescents. During the measurement of anthropometric weight and height data, calibration of the instrument was performed after each measurement. In addition, local food-colored pictures were used to minimize recall bias. The research lead investigator closely followed the day-to-day data collection process to ensure the completeness and consistency of the administered questionnaire.

### Statistical analysis

Statistical analysis was carried out using SPSS version 25 and WHO Anthro Plus software. A descriptive analysis was conducted to characterize the data using frequencies, percentages, means, and standard deviations. Before conducting further analysis, extreme values and the normality distribution of continuous variables were checked.

Dietary patterns were determined using exploratory factor analysis (EFA). Kaiser–Meyer–Olkin (KMO; value of *p* > 0.05) and Bartlett’s test of sphericity (value of *p*<0.05) were used to check the adequacy of the sample-to-factors ratio. The existence of a correlation between food items was determined for each step of factor analysis (29). Then, EFA was run under orthogonal rotation with the varimax method to select interpretable and independent dietary patterns. Factors with a communality above 0.5 and an eigenvalue above 1 explained by principal components were used to decide the final dietary patterns (30).

Nutritional outcomes of adolescents were determined based on height for age (HA), and body mass index (BMI) for age z-scores was computed using WHO AnthroPlus software (31, 32). A chi-square test was conducted to evaluate the effect of the identified dietary patterns on the burden of stunting and thinness of adolescents. Significant differences were determined with a value of *p* of < 0.05.

To identify factors for the identified dietary patterns, the first binary logistic regression analysis was carried out to measure the existence of an association between dietary patterns and factors. Then, factors that showed an association between binary logistic regression analysis and those with a *p*-value of <0.3 were fitted to the multivariable logistic regression model to identify significant factors. Significant factors were reported with a p-value of less than 0.05.

### Operational definition

Based on factor loadings, dietary patterns were determined using EFA. Based on the score, possible different dietary patterns were developed (33, 34). Then, each dietary pattern was classified into quartiles (Q) based on their contribution to each pattern, assuming an increasing order from Q1 to Q4 (35). Finally, Q1 and Q2 were combined to represent low consumption habit (0), while Q3 and Q4 represent high consumption of dietary habit (1) (35).

Adolescents’ nutritional status is determined based on height for age and body mass index for age using WHO AnthroPlus software (32). Then, HA z-scores < −2 SD were categorized as “stunted” and < −3 SD were categorized as “severely stunted,” and BMI for age z-scores < −2 SD was categorized as “thin” and > +1 SD was categorized as “overweight/obese” using the WHO cutoff points (31).

### Ethical standards of study

Ethical clearance was secured from the Institutional Review Board (IRB) of Addis Ababa University from the College of Natural and Computational Sciences. The study aims, possible risks, benefits, and privacy were described verbally to the study participants. Participation in the study was voluntary, and we gave them full autonomy to participate. To maintain confidentiality, the names and other identifiers of the study participants were not recorded in the data collection tool. Data on the anonymity and confidentiality of the study participants were assured. Informed verbal consent was obtained from parents of adolescents aged <18 years. Direct verbal informed consent was obtained from adolescents aged >18 years.

## Results

### Background characteristics

From the calculated sample size of 627 participants, 622 were involved in the study, with a response rate of 99.20%. The mean age of adolescents was 15.2 ± 2.02 years. More than half (60.10%) of the participants were girls. In total, 82.30% of adolescents’ mothers had no formal education, 11.90% attended elementary education, and the remaining 5.80% attended secondary education and above. Regarding the wealth index, 40.50% had lower thirds, 34.40% had medium thirds, and the remaining 25.10% had higher thirds (Table 1).

**Table 1:** Background characteristics of adolescents in Dembecha Woreda, Northwest Ethiopia, 2021.

Socio-demographic factors	Category of factors	Frequency (*n*)	Percentage (%)
Age of adolescents	10–13 years	26	4.2
	14–16 years	370	59.5
	17–19 years	226	36.3
Sex of adolescents	Male	248	39.9
	Female	374	60.1
Adolescents’ mothers’ educational level	No formal education	512	82.3
	Elementary school	74	11.9
	Secondary school and above	36	5.8
Wealth index of households	Lower thirds	252	40.5
	Medium thirds	214	34.4
	Higher thirds	156	25.1
Marital status of adolescents	Single	599	96.3
	Married	23	3.7
Family size	< 4 Members	209	33.6
	5–6 Members	292	46.9
	≥ 7 Members	121	19.5

### Dietary patterns of adolescents

The food lists were grouped into common food groups based on their similarity in nutrient contributions. Eight food groups were identified from the collected dietary consumption data. These food groups consist of traditional, legumes, tubers, whole grains, foods with high carbohydrate content, fruits and vegetables, animal sources, and traditional alcoholic beverages. Based on the EFA factor loading analysis, in the first component, traditional and legume diets had higher scores of 0.962 and 0.964, respectively. In the second component, fruit and vegetable diets, high carbohydrate (tea and white bread), and whole grain diets were identified with scores of 0.710, 0.653, and 0.519, respectively. In the third component, animal-source foods (eggs, meat, milk, and milk products) and traditional alcoholic beverages were identified with factor loadings of 0.746 and 0.625, respectively (Table 2).

**Table 2:** Exploratory factor analysis results of food frequency and factor loading consumed by adolescents in Dembecha Woreda, Northwest Ethiopia, 2021.

S. No.	Food list (item)	Type of dietary pattern	Components			H^2^
			1	2	3	
1	*Injera and Injera with Wot*	Traditional type	0.962*	0.017	0.014	0.938
2	Peas and Beans	Legume’s type	0.964*	0.020	0.020	0.383
3	Roasted wheat, barley, maize consumed as *kollo*, high-fiber sliced bread, and mixed-grain	Whole grain	0.262	0.519*	0.034	0.558
4	White bread, tea, and pasta	High Carbohydrate	−0.065	0.65*	0.310	0.326
5	Potato and sweet potato	Tuber’s type	0.048	0.409	−0.440	0.415
6	*Tela* (traditional home-fermented alcoholic beverage) and *areki or katicala* (traditional distilled alcoholic beverage)	Traditional alcoholic beverage	0.153	−0.134	0.625*	0.629
7	Eggs, meat, cooked meat, beef, lamb, milk, and milk products (butter, cheese, whole milk, and yogurt)	Animal-source foods	−0.095	0.201	0.746*	0.512
8	Banana, orange, pumpkin, carrot, fresh and cooked tomato, cabbage, green pepper, and lettuce	Fruits and vegetables, and	−0.059	0.710*	−0.228	0.931

H^2^ refers to communality, with items contributing significantly to each principal component analysis. *The food group score is the highest factor loading in each component. Bold values shows the highest factor loading of food group in each component.

**Figure 1:**
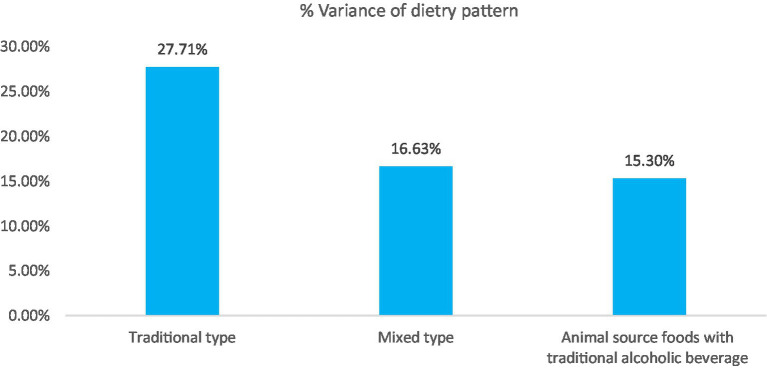
Percentage variance explained the three emerging dietary patterns of adolescents in Dembecha Woreda, Northwest Ethiopia, 2021.

Based on EFA analysis, three major types of dietary patterns were identified, which explained 58.64% of the variance in adolescent diets. The label assigned to each component was based on the items with high factor-loading scores and the interpretability of the factors. These include (1) the traditional type, explained by 26.71%; (2) mixed type, explained by 16.63%; and (3) animal-source foods with traditional alcoholic beverages, explained by 15.30% (Figure 1). From the identified dietary patterns, traditional, mixed, and animal-source dietary patterns were identified as healthy types; high-carbohydrate and traditional alcoholic beverage drinking patterns were identified as unhealthy types.

### Dietary patterns and its effect on nutritional outcomes

The prevalence of stunting was 26.30% (95% CL: 22.70, 30.20%), thinness was 9.60% (95% CL: 7.30, 12.40%), and overweight was 1.80%. Of the adolescents, 22.80% were moderately stunted, and the remaining 3.60% were severely stunted. This community-level undernutrition indicated that one in four and one in 10 adolescents were stunted and thin in their nutritional status, which became significant public health problems.

The burden of stunting was 15.12% versus 11.21 and 19.40% versus 6.94% for the lower and higher traditional and mixed dietary patterns, respectively. In contrast, 7.30% versus 19.00% of stunting was observed in higher and lower animal sources with alcoholic dietary patterns and consuming habits of adolescents, respectively (*p* < 0.05). However, there were no significant differences in thinness between the lower and higher consumption habits of the identified dietary patterns (*p*-value >0.05; Table 3).

**Table 3:** Dietary patterns and its effect on nutritional outcomes of adolescents in Dembecha Woreda, Northwest Ethiopia, 2021.

Types of dietary patterns	Nutritional status	Frequency of lower dietary score	Prevalence in lower dietary score	Frequency of high score	Prevalence in high dietary score	Pearson Chi-square	*P*-value
Traditional	Stunting	85	15.12%	63	11.21%	14.23	0.00001**
	Normal	163	29.00%	251	44.66%		
Mixed	Stunting	109	19.40%	39	6.94%	1.79	0.018**
	Normal	157	27.94%	257	45.73%		
Animal source with traditional alcoholic beverage	Stunting	41	7.30%	109	19.00%	9.13	0.003**
	Normal	173	30.80%	241	42.90%		
Traditional	Thinness	26	4.63%	28	4.98%	0.378	0.54
	Normal	218	38.79%	280	49.82%		
Mixed	Thinness	19	3.38%	35	6.23%	0.001	0.97
	Normal	174	30.96%	324	57.65%		
Animal source with a traditional alcoholic beverage	Thinness	20	3.40%	35	6.30%	0.17	0.83
	Normal	190	34.40%	308	55.80%		

**Significant (value of *p* < 0.05).

### Factors affecting the dietary patterns of adolescents

Adolescents who resided in low-land agroecology (AOR = 2.4; 95% CL: 1.24, 4.81) and mid-land agroecology (AOR = 0.48; 95%: 0.31, 0.74), the early age range of 10–13 years (AOR = 0.6; 95% CL: 0.46, 0.99), and had access to animal sources (AOR = 1.64; 95% CL: 1.04, 2.60) were significantly associated with a lower consumption habit of traditional dietary pattern (Table 4).

A mixed type of dietary pattern was the second component of a dietary pattern, which was explained by 16.63% of all dietary patterns. Among sociodemographic factors, adolescents who were male (AOR =2.28; 95% CL: 1.56, 3.34), resided in mid-land agroecology (AOR = 1.80; 95% CL: 1.18, 2.74), high-land agroecology (AOR = 2.14; 95% CL: 1.19, 3.87), whose mothers had no formal education (AOR = 2.38; 95% CL: 1.35, 4.19), lived with large family size (AOR = 1.69; 95% CL: 1.02, 2.80), and had poor nutritional knowledge (AOR = 2.83; 95% CL: 1.12, 4.05) had a significant association with lower consumption of a mixed type of dietary pattern (Table 4).

**Table 4:** Contributing factors for lower consumption of major dietary patterns among adolescents, Dembecha Woreda, Northwest Ethiopia, 2021.

Variables	Variable category	Traditional type			*p*-value	Mixed dietary pattern			*p*-value	Animal-source foods and traditional alcoholic beverage patterns			*p*-value
		AOR				AOR				AOR			
		AOR	LC	UL		AOR	LC	UL		AOR	LC	UL	
Sex of adolescent	Male	1	1	1		2.28**	**1.56**	**3.34**	0.0001**	1	1	1	
	Female	1.32	0.89	1.95	0.12		1			1.87**	**1.27**	**2.74**	0.0001**
Agroecology	Low-land	**2.44**	**1.24**	**4.81**	0.01**	1.80**	**1.18**	**2.74**	0.01**	1	1	1	
	Mid-land	**0.48**	**0.31**	**0.74**	0.001**	2.14**	**1.19**	**3.87**	0.01**	1.47	0.96	2.25	0.7
	High-land	1	1	1		1	1	1		2.50**	**1.37**	**4.56**	0.0001**
Age in years	10–13	**0.67**	**0.46**	**0.99**	0.045**	1	1	1		1.77	0.70	4.52	0.23
	14–16	1.01	0.42	2.41	0.99	0.99	0.41	2.38	0.98	0.91	0.35	2.37	0.85
	17–19	1	1	1		1.09	0.75	1.59	0.64	1	1	1	
Adolescents’ mothers’ level of education	No formal education	1	1	1		2.38**	**1.35**	**4.19**	0.001**	0.54	0.26	1.12	0.10
	Elementary level	0.95	0.41	2.17	0.89	0.64	0.30	1.39	0.26	0.42	0.17	1.02	0.06
	Secondary and above	1.00	0.39	2.58	0.99	1	1	1		1	1	1	
Wealth index	Lower thirds	1	1	1		1.11	0.69	1.78	0.67	0.81	0.54	1.21	0.31
	Medium thirds	0.63	0.39	1.03	0.23	1.05	0.65	1.68	0.84	0.62	0.38	1.01	0.05
	Higher thirds	0.72	0.44	1.17	0.16	1	1	1		1	1	1	
Family Size	< 4 Member	1	1	1		1	1	1	0.14	1	1	1	
	5–6 Member	0.94	0.56	1.60	0.83	1.44	0.89	2.32		0.68	1.87	0.64	0.64
	> 7 Member	0.74	0.45	1.22	0.24	1.69**	**1.02**	**2.80**	0.04**	0.79	2.03	0.33	0.33
Nutrition knowledge	Poor	0.69	0.38	1.26	0.23	2.83**	**1.55**	**5.19**	0.001**	0.82	2.62	0.19	0.19
	Medium	0.66	0.37	1.17	0.16	2.90**	**1.60**	**5.28**	0.01**	0.56	1.74	0.97	0.97
	Good	1	1	1		1	1	1		1	1	1	
Irrigation land access	Yes	1.22	0.83	1.79	0.32					0.6 8**	**0.47**	**0.99**	0.04**
	No	1	1	1		1.29	0.89	1.87	0.18	1	1	1	
Getting nutrition education	Yes	1.51	0.78	2.92	0.23	1	1	1		1	1	1	
	No	1	1	1		2.13**	**1.12**	**4.05**	0.02**	0.92	0.50	1.69	0.79
Access to animal-source food	Yes	**1.64**	**1.04**	**2.60**	0.03**	1	1	1		1	1	1	
	No	1	1	1		0.97	0.68	1.37	0.84	0.87	0.56	1.36	0.54
Access to vegetables and fruits	Yes	1.20	0.83	1.74	0.33	1	1	1		1.20	0.85	1.71	0.30
	No	1	1	1		0.97	0.68	1.37	0.67	1	1	1	

**Significant factors (*p*-value < 0.05); AOR-Adjusted odd ratio; LC-lower confidence level; UL-upper confidence level. Bold values indicates significant factors with value of less than 0.05.

A third type of dietary habit was explained by a 15.30% variance. This dietary pattern was characterized by the consumption habits of animal-source foods (milk, eggs, and meat) with traditional alcoholic drinking. Traditional alcohol consumption is an unhealthy dietary pattern. Overall, this type of dietary pattern lacks vitamins and minerals that are found in vegetables and fruits. Being female adolescents (AOR = 1.87; 95% CL: 1.27, 2.74), those who were residing in high-land agroecology (AOR = 2.50; 95% CL: 1.37, 4.56) and had access to irrigation land (AOR = 0.68; 95% CL: 0.47, 0.99) had a significant association with lower consumption of animal sources with traditional alcoholic beverage dietary patterns (Table 4).

## Discussion

Three main types of dietary patterns were identified, of which traditional, mixed, and animal-sourced foods were considered healthy types. However, high-carbohydrate and traditional alcoholic beverages were unhealthy. The identified dietary patterns were the most common and easily accessible in the local area through agricultural production in developing countries (12). This identified healthy and unhealthy dietary habits of adolescents were practiced among adolescents in developing countries such as Ghana (2) and India (36).

In the current study, adolescents had lower consumption habits of healthy dietary patterns of fruits and vegetables. This finding is similar to the study conducted among urban adolescents in Bangladesh, where their habitual dietary pattern indicated poor consumption of leafy vegetables (33). This feeding habit predisposes adolescents to essential micronutrient deficiencies (33, 37).

Overall, 26.30 and 9.60% of adolescents were stunted and thin in their nutritional outcomes. These findings indicate that undernutrition among adolescents is a public health concern in the study setting. The burden of stunting was 15.12% versus 11.21, 19.40% versus 6.94, and 8.36% versus 17.97% in adolescents with lower traditional, mixed, and higher animal sources with alcoholic dietary consumption habits, respectively. This is because the effect of lower consumption dietary pattern does not satisfy the daily requirement of nutrients for the physical growth and development of adolescents (21). Inadequate intake of macronutrients and micronutrients, poor quantity, and quality dietary habits lead to undernutrition (6, 37, 38).

Regarding animal sources with the traditional alcoholic dietary pattern, the prevalence of stunting was 7.30% in the lower consumption group and 19.00% in the higher consumption group. This diet is characterized by the feeding habits of food items such as meat, eggs, milk, and milk products, along with traditional alcoholic drinking. However, the effects of alcohol and its metabolism prevent the absorption and utilization of nutrients. Hence, it leads to a deficiency of micronutrients and macronutrients, leading to undernutrition (39).

There were no significant differences in thinness between adolescents with lower and higher scores in the three identified dietary patterns. Thinness among adolescents was not affected by long-term dietary habits but rather by immediate factors such as infections (40, 41), diversification in feeding practice (41), and household food insecurity (42, 43).

The second type of dietary pattern was identified as the mixed type. Of all adolescents, one-fourth (25.5%) consumed a whole-grain diet per week. This is considered a healthy type due to the nutritional contribution of vitamins and minerals, which are found in fruits and vegetables. In addition, a whole-grain-based diet can provide energy, starch, and dietary fiber (44–46). A similar pattern was identified in a study conducted among school-age children in Scandinavian countries (47) and Ghana (12).

Among sociodemographic factors, adolescents who live with large family sizes had two times lower consumption habits of mixed types of dietary patterns. This is due to the effect of large family influence on the economic insufficiency of households to meet their diversified dietary needs (48, 49). A similar study confirmed that the dietary patterns of adolescents were significantly determined by the socioeconomic status of a family (50). Adolescents who belonged to low socioeconomic status did not consume healthier diets as compared with those of middle and high socioeconomic status (49, 51).

Adolescents’ mothers who did not attend formal education were two times more likely to have lower consumption of mixed types of dietary patterns. As the educational status of adolescents’ mothers improved, they had a chance to get information on healthy dietary habits to translate nutritional knowledge into practice (52, 53).

Among modifiable factors, adolescents who had poor nutrition knowledge and lack of exposure to nutrition education were three and two times more likely to have a lower consumption habit of mixed types of dietary patterns. This might be due to the effect of poor nutritional knowledge on the mixed feeding habits of adolescents (49). Furthermore, as adolescents have sufficient basic nutritional knowledge, they can get enough information about the nutrient contents of diversified food items (49, 54, 55).

The third dietary pattern was mainly characterized by the consumption of milk, meat, and eggs, along with the drinking habit of traditional alcoholic beverages. From the animal-source foods, sea foods such as fish were not consumed in the study area due to a lack of access in the study setting (56, 57).

Female adolescents were nearly two times more likely to consume low-animal-source foods and alcohol as compared with their male counterparts. This is due to community social norms in intra-household food serving as staple food items are distributed fairly equally, and side dishes usually containing animal-source food (like meat, eggs and, milk) are provided for male adolescents (58).

In addition, evidence in developing countries indicates that at the household level, staple food items are distributed fairly to all family members, and side dishes usually containing more micronutrients (such as meat) are often preferably allocated to male heads of household and male children. This brings lower consumption habits for animal-source foods among adolescent girls (59, 60).

## Strengths and limitations of the study

This is a comprehensive study that assesses the dietary patterns of adolescents and their effect on nutrition outcomes in the study setting. The study focused on adolescents, who were understudied and overlooked population group in nutrition interventions in developing countries. As a limitation, the inability to identify all food items that are used to prepare the local traditional diet, as adolescents were not involved in cooking at home, might lead to under-reported food items such as spices.

## Conclusion

Generally, the dietary pattern of adolescents is dominated by a plant-based diet with limited consumption of micronutrient-rich sources of nutrition. From the identified dietary patterns, traditional, mixed, and animal-source dietary patterns were identified as healthy types; high carbohydrate and traditional alcoholic beverage drinking patterns were identified as unhealthy types. Significantly, the burden of stunting was relatively higher among adolescents who had lower consumption habits of traditional and mixed dietary patterns and higher consumption habits of animal sources with alcoholic diets. However, there were no significant differences in thinness between lower and higher consumption habits in the identified dietary pattern.

Multidimensional modifiable factors were identified for lower consumption of healthy dietary patterns, which could be targeted for public health interventions. Integrated and multifaceted dietary intervention approaches are needed to promote a healthy diet, discouraging the consumption of unhealthy diets to reduce the burden of stunting in the study area and similar settings. Enhancing the sea-food consumption habits of adolescents and the entire community by introducing small-scale fish farms as nutrition-sensitive intervention pilot projects in study settings and beyond.

## Data availability statement

The original contributions presented in the study are included in the article/supplementary materials, further inquiries can be directed to the corresponding author/s.

## Author contributions

EA: Conceptualization, Data curation, Formal analysis, Investigation, Methodology, Software, Supervision, Writing – original draft, Writing – review & editing. ZA: Conceptualization, Methodology, Supervision, Validation, Writing – review & editing. AA: Investigation, Methodology, Supervision, Validation, Writing – review & editing.

## Abbreviations

AOR: Adjusted odd ratio
BMI: Body mass index
EEA: Exploratory factor analysis
HA: Height for age
FFQ: Food frequency questionnaire
IDA: iron deficiency anemia
IDD: iodine deficiency disorders
MNDs: Micronutrient deficiencies
VAD: vitamin-A deficiency
WHO: World Health Organization
